# Case report: absence of the right piriformis muscle in a woman

**DOI:** 10.1007/s00276-018-02176-6

**Published:** 2019-02-13

**Authors:** Erich Brenner, Massimiliano Tripoli, Elia Scavo, Adriana Cordova

**Affiliations:** 1 Division of Clinical and Functional Anatomy (Director: o.Univ.Prof. Dr. H. Fritsch), Medical University of Innsbruck, Muellerstrasse 59, 6020 Innsbruck, Austria; 2 Chirurgia Plastica Ricostruttiva ed Estetica, Policlinico ‘Paolo Giaccone’ di Palermo, Palermo, Italy; 3 Department of Surgical Oncological and Oral Science (DiChiRonS), Palermo University, Palermo, Italy

**Keywords:** Piriformis muscle, Anatomical variation, Deep gluteal region, Common gluteal artery, Gemellus inferior muscle, Anatomical variation

## Abstract

We report a very rare case of a unilaterally absent piriformis muscle in a 60 year old woman. Accompanying variations comprised a common gluteal artery (instead of two distinct superior and inferior gluteal arteries), and an absent gemellus inferior muscle. The contralateral left side showed a normally developed piriformis muscle. In hominoids, the piriformis is constant, but is regularly missing in several other vertebrates. The piriformis muscle is an anatomical landmark for ultrasound investigations and ultrasound-guided interventions in the deep gluteal region such as a superior gluteal nerve block or even a sacral plexus block, also for any surgical approach such as total hip arthroplasty. A missing piriformis muscle therefore affects the orientation in the deep gluteal region and therefore the identification of the targeted structures.

## Introduction

The piriformis muscle is clinically well known for the compression of the sciatic nerve, the piriformis (muscle) syndrome or deep gluteal syndrome [11].

Related on this pathology, a vast amount of studies investigated the relationship between the piriformis muscle and the sciatic nerve, reporting the variability of the piriformis muscle mainly according to the classification by Beaton and Anson [3]. None of these studies reported an absent or missing piriformis muscle (e.g. [18]). Also fetal studies found a constant piriformis muscle (e.g. [2]).

The literature provides only few reports on a missing piriformis muscle. Alexander Macalister found one case and reported this very case at least three times (e.g. Ref. [9]). Furthermore, Macalister cites case reports by Otto and Budge: in the subject dissected by Budge the lower limb was deformed [9], and in the specimen dissected by Otto the gemellus superior was very large [9]. These last observations are not sufficiently known for us to judge whether it was actually a real absence of the piriformis muscle or simply the fusion with one of the neighboring muscles, such as the gemellus superior or the gluteus medius muscles. The extensive compilation on anatomical variations of the piriformis muscle by Nicholson et al. [12] reports erroneously a paper, which should have described two missing piriformis muscles out of six specimens; nevertheless, that paper [5] describes six piriformis muscles in six specimens, whereas the gemellus superior was missing in two, and the gemellus inferior in one case.

## Case report

We report here the case of a 60-year old Caucasian woman who died from amyotrophic lateral sclerosis. She donated her body to the anatomical department giving informed consent for using her body for scientific and educational purposes prior to death [14]. At admission a 30 cm scar was found at the left lateral thigh, and another 10 cm transversal collar scar. No further medical data are available. Her corpse was preserved using an arterial injection of a formaldehyde–phenol solution and immersed in phenolic acid in water for 3 months [13].

The corpse was used for a surgical-anatomical study on the superficial and subfascial vascularity of the gluteal region. When dissecting the right side and detaching the gluteus maximus muscle to display the gluteal arteries and veins no piriformis muscle could be found.

### Description

**Fig. 1:**
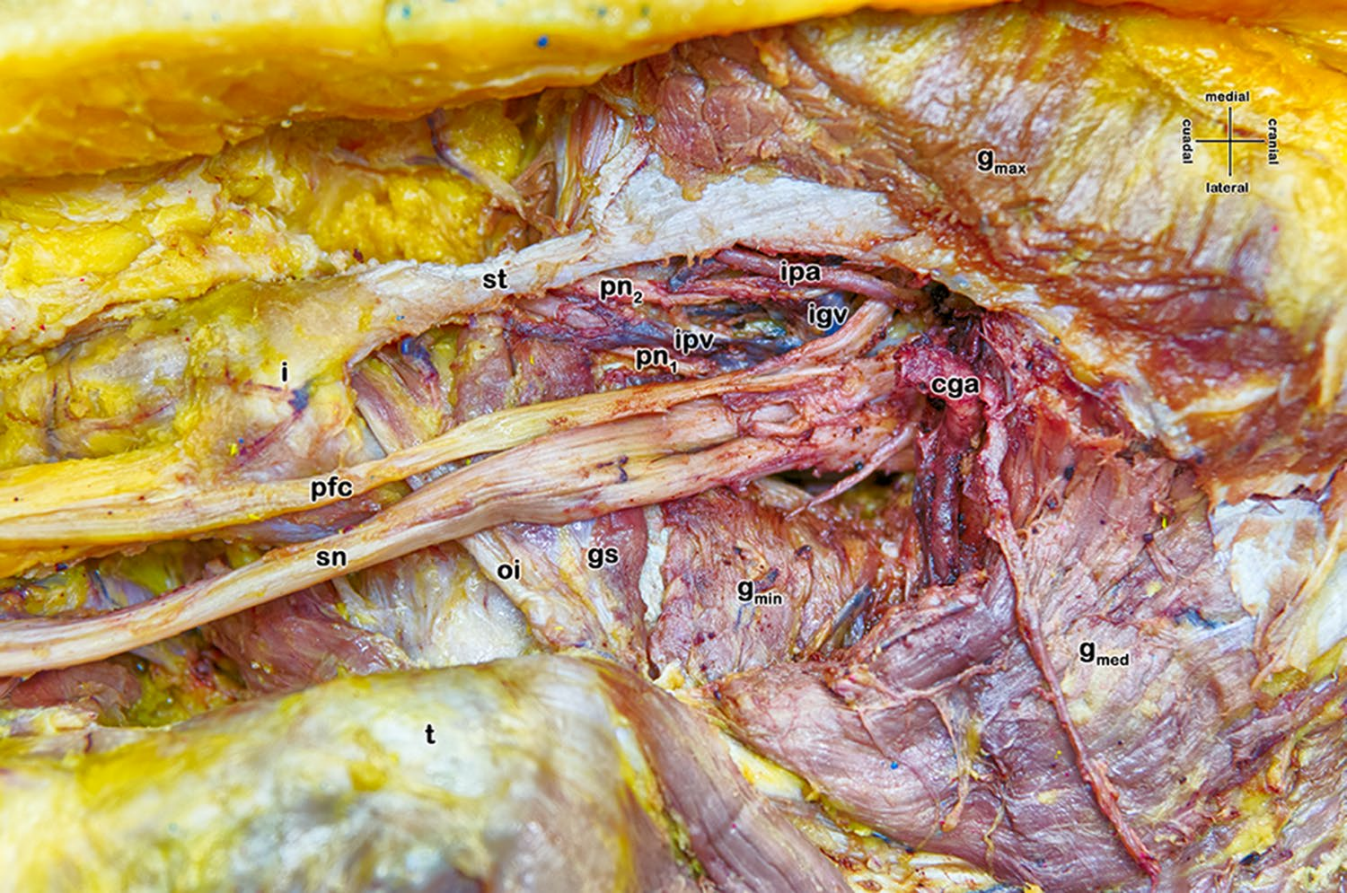
Right deep gluteal region with absent piriformis muscle (female, 60 years)

The greater sciatic foramen is properly formed by the greater sciatic notch, the sacrotuberal and the sacrospinal ligaments. Only neurovascular structures pass, a common gluteal artery (replacing the superior gluteal artery), a superior gluteal vein, the sciatic nerve, an inferior gluteal vein, a (bipartite) pudendal nerve and the internal pudendal vessels. In other words, the piriformis muscle is missing as well as the inferior gluteal artery. Both, a vessel resembling the ‘descending branch of the inferior gluteal artery’ and the artery to the sciatic nerve originate from the common gluteal artery. Furthermore, at the lesser sciatic foramen, a quite large gemellus superior muscle accompanies the obturator internus muscle, whereas the gemellus inferior muscle is also missing (Fig. 1).

The left side shows no variations; the piriformis muscle exists.

## Discussion

As this case seems to be the second ‘verified’ throughout literature we have to discuss carefully possible probabilities such as (complete) fusion with another muscle, the (complete) involution or atrophy due to a medical intervention, or a real aplasia.

### Fusion with another muscle

The piriformis muscle might fuse more or less completely with the gluteus medius, the gluteus minimus or the gemellus superior muscles, respectively. None of these is the case here.

### Involution due to a medical intervention

Medical interventions for the treatment of piriformis muscle syndrome include methods such as botulinum toxin injections [1], surgical or endoscopic transection, i.e., tenotomy [8], or rupture during total hip arthroplasty [15]. Al-Al-Shaikh et al. [1] showed that tenotomy of the piriformis muscle leads to atrophy and fatty infiltration, but not to a complete disappearance. Furthermore, other authors argue that tenotomy might even increase the symptoms of piriformis muscle syndrome (post-tenotomy sciatica) due to retraction of the piriformis muscle [17].

### Real aplasia

This is the most probable reason.

### Clinical relevance

The piriformis muscle is an anatomical landmark for ultrasound investigations and ultrasound-guided interventions in the deep gluteal region such as a superior gluteal nerve block or even a sacral plexus block. Furthermore, the piriformis muscle is an important guide for any posterior surgical approach such as total hip arthroplasty. A missing piriformis muscle therefore affects the orientation in the deep gluteal region and therefore the identification of the targeted structures. Of course, the diagnosis of a piriformis syndrome and related interventions would be obsolete in a further patient with an absent piriformis muscle.

### Comparative anatomy

In all hominoids the piriformis originates from the anterior surface of the sacrum, as well as from its lateral aspect, and inserts onto the greater trochanter of the femur [7]. In *Babirusas*, also called deer-pigs, this muscle has fused completely with the M. gluteus medius [10]. The piriformis of human anatomy differs in origin from that of the elephant, where it arises dorso-laterally from the sacral vertebrae close to the tuber sacrale of the ilium, and blends both with the superficial and middle glutei [6]. Based on the literature, the piriformis muscle is absent in several vertebrates, such as solipeds, ruminants, porks, macaques, spider monkeys, coati, bats, etc. [16]. The deep gluteal anlage, i.e., the gluteus medius, gluteus minimus and piriformis, is clearly homologous to the lizard iliofemoralis because in both groups this anlage has an acetabular bony origin, a proximal insertion, and a position posterior to the fibular nerve trunk [4].

## Abbreviations

gi: Gemellus inferior muscle
gs: Gemellus superior muscle
tg_max_: Gluteus maximus muscle
g_med_: Gluteus medius muscle
g_min_: Gluteus minimus muscle
igv: Inferior gluteal vein
ipa: Internal pudendal artery
ipv: Internal pudendal vein
oi: Obturator internus muscle
pm: Piriformis muscle
pfc: Posterior femoral cutaneous nerve
pn_1_, pn_2_: Doubled pudendal nerve
st: Sacrotuberal ligament
sn: Sciatic nerve
cga: Common gluteal artery
sgv: Superior gluteal vein
t: Greater trochanter
i: Ischial tuberosity
